# Cytokeratin-positive interstitial reticulum cell (CIRC) tumor in the lymph node: a case report of the transformation from the epithelioid cell type to the spindle cell type

**DOI:** 10.1186/s13000-020-01032-9

**Published:** 2020-09-26

**Authors:** Sachiko Kaji, Nobuyuki Hiruta, Daisuke Sasai, Makoto Nagashima, Rintaro Ohe, Mitsunori Yamakawa

**Affiliations:** 1 Department of Diagnostic Pathology, Chiba Kaihin Municipal Hospital, 3-31-1 Isobe, Mihama-ku, Chiba, 261-0012 Japan; 2 Department of Surgical Pathology, Toho University Sakura Medical Center, Sakura, Japan; 3 Department of Pathology, Seirei Sakura Citizen Hospital, Sakura, Japan; 4 Department of Surgery, Toho University Sakura Medical Center, Sakura, Japan; 5 Department of Pathological Diagnostics, Yamagata University Faculty of Medicine, Yamagata, Japan

**Keywords:** Cytokeratin-positive interstitial reticulum cells, Fibroblastic reticular cells, Cell morphology, Transform, Autopsy

## Abstract

**Background:**

Cytokeratin-positive interstitial reticulum cells (CIRCs), which are a subgroup of fibroblastic reticular cells (FRCs), are known to be present in the lymph nodes. There have been only a few cases of tumors derived from CIRCs.

**Case presentation:**

We have reported a new case involving a CIRC tumor in a 75-year-old man and reviewed the literature. The resected mediastinal lymph nodes showed epithelial-like proliferation of large atypical round and polygonal epithelioid cells. The tumor cells expressed CK8, CK18, CAM5.2, AE1/AE3, epithelial membrane antigen, vimentin, fascin, and some FRC markers, which is consistent with the diagnosis of a CIRC tumor. Following chemotherapy, the CIRC tumor was observed to have responded very well and became difficult to confirm on imaging, but a small cell lung carcinoma developed 12 months later. Chemoradiotherapy was performed, but the patient passed away 29 months after the initial diagnosis. The autopsy revealed the recurrence of the CIRC tumor, residual small cell lung carcinoma, and a very small latent carcinoma of the prostate. The relapsed CIRC tumor cells had a spindle shape; they were highly pleomorphic and had invaded the superior vena cava.

**Conclusion:**

We first reported autopsy findings of CIRC tumors and demonstrated the transformation of the tumor from the epithelioid cell type to the spindle cell type.

## Background

Lymph node structure and function are supported by dendritic/reticular cells, which are divided into three subtypes: follicular dendritic cells (FDCs), interdigitating dendritic cells (IDCs), and fibroblastic reticular cells/fibroblastic reticulum cells (FRCs). FRCs are located in the parafollicular and deep cortex areas and comprise a mesh-like reticular network with reticular fibers and fibrous extracellular matrix bundles [1–6]. FRCs are embedded in the extracellular matrix [7] and are in contact with immune cells [3]. A subgroup of FRCs that can express cytokeratins are called cytokeratin-positive interstitial reticulum cells (CIRCs). CIRC tumors are considered a subset of FRC tumors.

CIRC tumors are difficult to diagnose because of their nature. The expression pattern of CIRC tumors is a challenge for pathologists, as several neoplasms express cytokeratins and vimentin, which can result in confusion. It is important to distinguish metastatic carcinoma, Hodgkin lymphoma, FDC sarcoma, and IDC sarcoma. Very few reports of cases of CIRC tumors have been published [8–14]; among these cases, only two had a fatal clinical outcome [9, 11]. To the best of our knowledge, this is the first report of a case of a CIRC tumor with autopsy findings. The autopsy provided evidence of the biological behavior of the CIRC tumor.

We first report here a new case involving a CIRC tumor in a 75-year-old man and transformation of the tumor from the epithelioid cell type of a primary lymph node tumor to the spindle cell type of a recurrent tumor on autopsy. The present study has provided specific immunohistochemical findings as well as electron microscopy findings for this tumor; in addition, we have reviewed the literature.

## Case presentation

### Clinical presentation

**Fig. 1:**
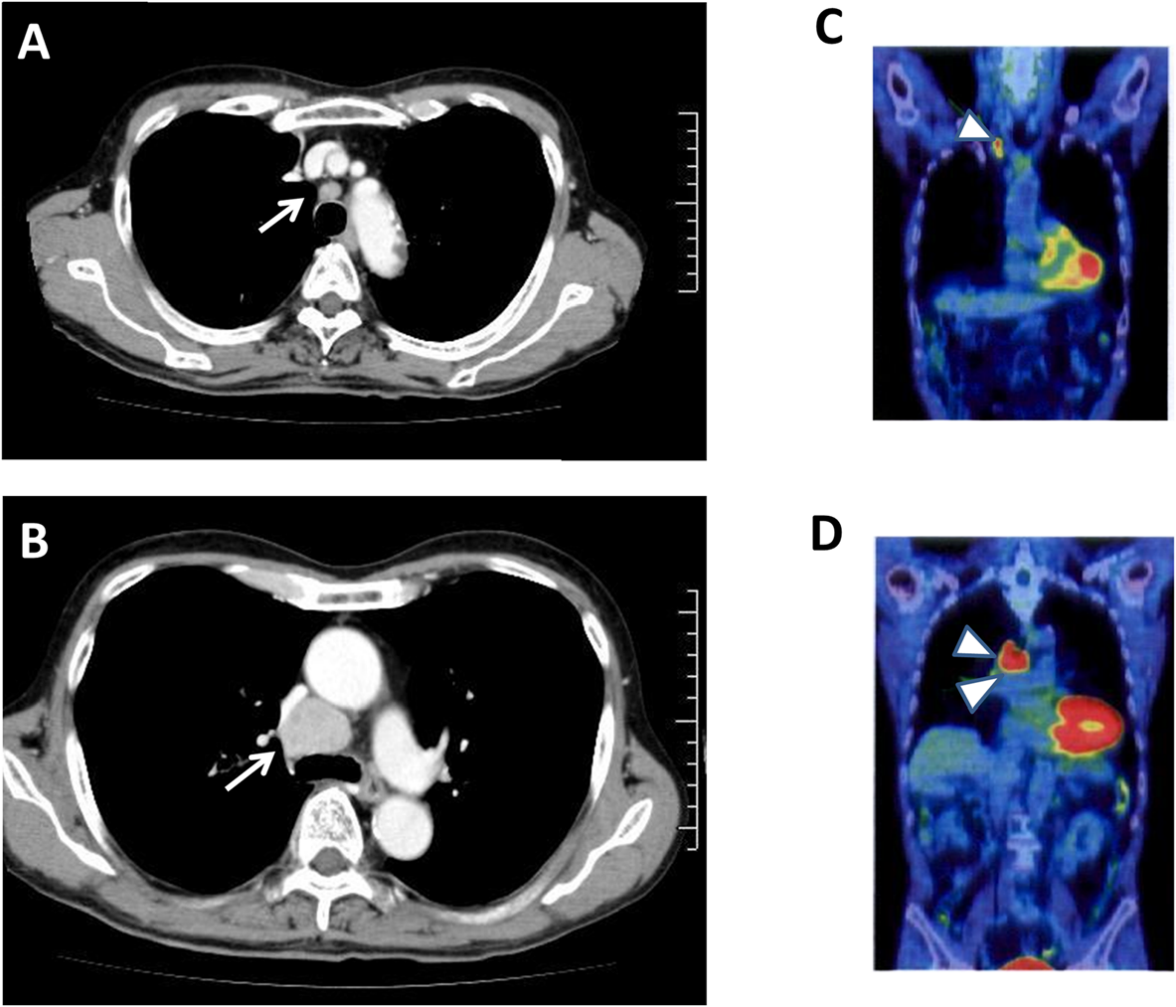
Enhanced computed tomography revealed swelling of the right upper paratracheal lymph node (**a**, *arrow*) and right lower paratracheal lymph node (**b**, *arrow*). Abnormally high uptake in the right supraclavicular lymph node (**c**, *arrowhead*) and right lower paratracheal lymph node (**d**, *arrowheads*) was detected by positron emission tomography/computed tomography. The right lower paratracheal lymph node was the main lesion, which was approximately 2.7 cm in size. The right upper paratracheal lymph node and right supraclavicular lymph node were metastatic lesions and showed slight swelling

A 75-year-old Japanese man was found to have mediastinal lymphadenopathy by computed tomography (Fig. 1a, b). Transbronchial aspiration cytology was performed at the Sakura Medical Center, Toho University. The cytological diagnosis confirmed that it was a malignant tumor with the possibility of it being a lymphoreticular tumor or a metastatic carcinoma. Three months later, a lymphadenectomy was performed. The histological findings suggested that the tumor was a malignant lymphoma and was most likely Hodgkin lymphoma or a metastatic carcinoma. Immunohistochemically, the tumor cells expressed cytokeratins and vimentin and were negative for lymphoid markers. These findings suggested that the tumor was likely a metastatic carcinoma. However, positron emission tomography/computed tomography showed no other lesions throughout the body except for swollen mediastinal lymph nodes (Fig. 1c, d), and gastrointestinal endoscopic examinations revealed no other abnormalities. The levels of serum tumor markers, including carcinoembryonic antigen, carbohydrate antigen 19–9, squamous cell carcinoma antigen, neuron specific enolase (NSE), progastrin-releasing peptide, and cytokeratin 19 fragment, were within the normal ranges. The serum soluble interleukin-2 receptor level was not at a high level (469 U/ml). Based on this result, a CIRC tumor was suspected.

The CIRC tumor responded very well to chemotherapy and became difficult to confirm on imaging, but 12 months later, a new tumor was detected in the hilar region of the right lung on computed tomography. A small cell carcinoma was diagnosed by transbronchial aspiration cytology. The patient underwent chemoradiotherapy, but he later presented with superior vena cava syndrome and passed away. The total clinical course lasted for 29 months.

### Pathological findings

#### Lymph node findings

**Fig. 2:**
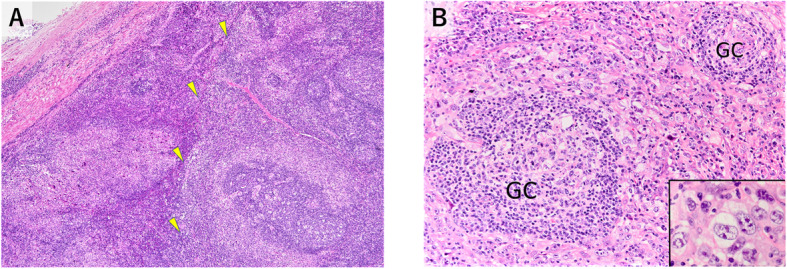
Histology of the primary cytokeratin-positive interstitial reticulum cell (CIRC) tumor of the biopsied lymph node. **a** The nodal architecture showed effacement caused by neoplastic proliferation on the left side. Circumferential tumor cell proliferation around follicles was conspicuous on the right side. The tumor margin was vaguely detectable (*arrowheads*). **b** Proliferating tumor cells around the germinal center (*GC*) were large in size and round or polygonal. Most of them had round vesicular nuclei and prominent nucleoli. The abundant cytoplasm was pale to eosinophilic. Tumor cells were admixed with many small lymphocytes, plasma cells and eosinophils. ***Inset***: high-magnification view of the tumor cells

The completely excised mediastinal lymph node measured approximately 2.7 × 2.0 × 2.1 cm. On microscopic evaluation, the nodal architecture was mostly preserved, except in areas where neoplastic proliferation caused partial effacement (Fig. 2a). The lymph node was inconspicuously demarcated by fibrous tissue continuing to the capsule. Circumferential tumor cell proliferation around the follicles was conspicuous (Fig. 2a, b). Tumor cells were highly atypical, large in size, and round or polygonal in shape. Most of the tumor cells had round vesicular nuclei and prominent nucleoli. The abundant cytoplasm was pale to eosinophilic, with an ill-defined cell border resulting in an epithelioid-like appearance (Fig. 2b). The tumor cells were admixed with many small lymphocytes, plasma cells, and eosinophils. Tumor cells that resembled Hodgkin’s cells and Reed–Sternberg cells were scattered throughout the tumor. Typical and atypical mitoses were frequent (20/10 high-power fields). Apoptotic cells were also noted. Delicate collagen fibers often intermingled with tumor cells. The tumor cells and collagen fibers were closely associated. The cytoplasm of some tumor cells was hyalinized and appeared to continue to form collagen fibers. The tumor cells were often elongated in areas of increased numbers of collagen fibers. Necrotic foci and hemorrhage were not observed. Metastases of two small mediastinal lymph nodes were recognized.

**Fig. 3:**
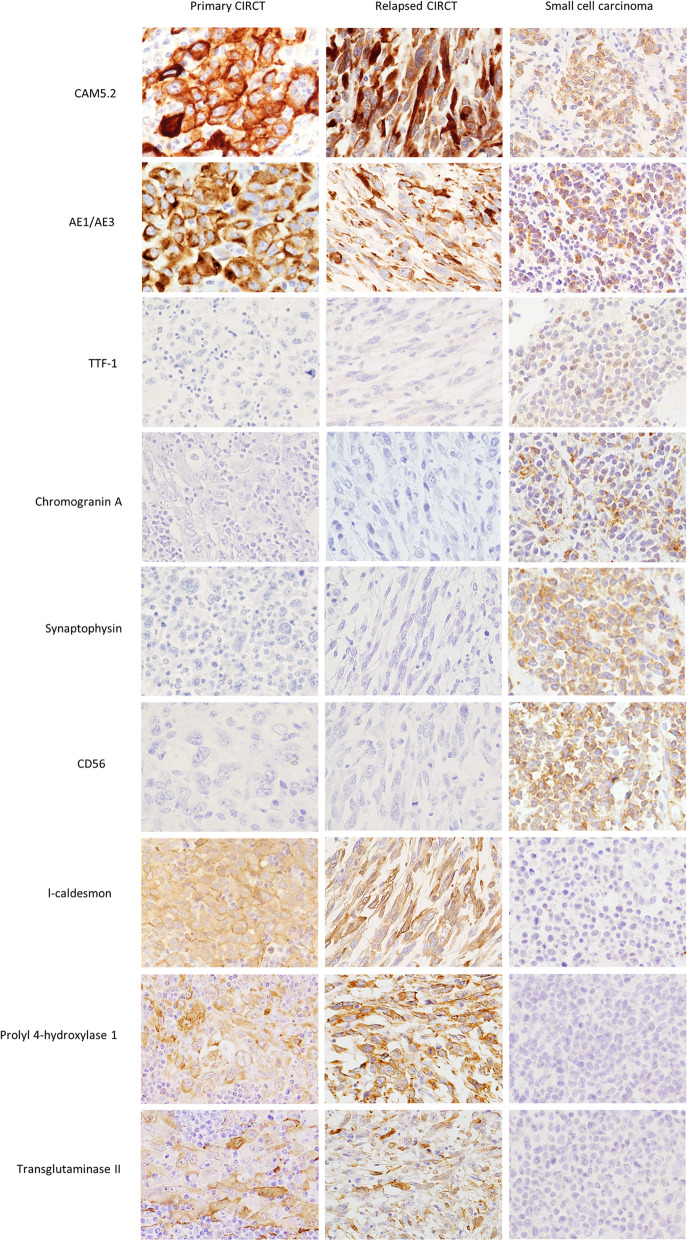
Immunohistochemical staining of primary cytokeratin-positive interstitial reticulum cell (CIRC) tumors, relapsed CIRC tumors and small cell lung carcinoma using CAM5.2, AE1/AE3, TTF-1, chromogranin A, synaptophysin, CD56, l-caldesmon, prolyl 4-hydroxylase 1 and transglutaminase II antibodies. Tumor cells of primary CIRC tumors and relapsed CIRC tumors were positive for CAM5.2 and AE1/AE3 and negative for TTF-1. Tumor cells of small cell lung carcinoma were focally positive for CAM5.2 and AE1/AE3 and weakly positive for TTF-1. Tumor cells of primary CIRC tumors and relapsed CIRC tumors were negative for chromogranin A, synaptophysin and CD56. Tumor cells of small cell lung carcinoma were focally positive for chromogranin A and strongly positive for synaptophysin and CD56. Tumor cells of primary CIRC tumors and relapsed CIRC tumors were positive for l-caldesmon, prolyl 4-hydroxylase 1 and transglutaminase II. Tumor cells of small cell lung carcinoma were negative for l-caldesmon, prolyl 4-hydroxylase 1 and transglutaminase II

The tumor cells were immunohistochemically positive for cytokeratin (CK)7, CK8, CK18, CAM5.2, AE1/AE3, epithelial membrane antigen (EMA), vimentin, fascin, l-caldesmon, prolyl 4-hydroxylase 1, lysyl hydroxylase 3, and transglutaminase II (Table 1, Fig. 3). Immunoreaction with cytokeratins revealed fine cytoplasmic projections and reticular patterns in the tumor cells. Immunohistochemical staining of tenascin-C, prolyl 3-hydroxylase 1, alpha smooth muscle actin (α-SMA), desmin, FDC markers (CD23, D2–40, clusterin, CNA.42, low affinity NGFR, CD21, and CD35) and histiocytic/dendritic cell markers (CD1a, CD11c, langerin, DC-SIGN, DC-LAMP, CD83, DEC205, S-100 protein, CD68, and Factor XIIIa) were negative. In addition, the staining of other lymphocyte markers, including CD3, CD4, CD5, CD8, CD20, CD30, CD45, CD45RO, CD79a, and PAX5, were all negative. Staining of markers important for distinguishing metastatic tumors (34βE12, prostate-specific antigen, NKX3.1, P504S, polyclonal carcinoembryonic antigen, CK5/6, CK20, TTF-1, Napsin A, p63, p40, Melan A, HMB45, CD34, CD117, and ALK) were negative. Staining of neuroendocrine markers (NSE, chromogranin A, synaptophysin, and CD56) were also negative. Detection of Epstein-Barr encoding region (EBER)-1 was negative by in situ hybridization (ISH). Less than 10% of the tumor cells showed weak expression of EGFR. Up to 95% of the tumor cells showed high expression of the p53 protein. The Ki-67 labeling index (LI) was high (greater than 75%).

**Table 1:** Immunophenotypes of CIRC tumor and small cell carcinoma of this case

Antibody	Immunophenotype		
	CIRC tumor		Small cell carcinoma
	Primary tumor	Relapsed tumor	
***Epithelial markers***			
CK7	+	–	–
CK8	+	+	±
CK18	+	+	±(weakly)
CAM5.2	+	+	±
AE1/AE3	+	+	±
EMA	+	+	+
34βE12	–	–	–
PSA	–	–	–
NKX3.1	–	–	–
P504S	–	–	–
pCEA	–	–	–
CK5/6	–	–	–
CK20	–	–	–
TTF-1	–	–	±(weakly)
Napsin A	–	–	–
p63	–	–	–
p40	–	–	–
***Neuroendocrine cell markers***			
NSE	–	–	+
Chromogranin A	–	–	±
Synaptophysin	–	–	+
CD56	–	–	+
***Lymphoid markers***			
CD3	–	–	–
CD4	–	–	–
CD5	–	–	–
CD8	–	–	–
CD20	–	–	–
CD30 (Ki-1)	–	–	–
CD45 (LCA)	–	–	–
CD45RO (UCHL-1)	–	–	–
CD79a	–	–	–
PAX5	–	–	–
EBER-1*	–	–	–
***FRC markers***			
Tenascin-C	–	–	–
Prolyl 3-hydroxylase 1	–	–	–
l-caldesmon	+	+	–
Prolyl 4-hydroxylase 1	+	+	–
Lysyl hydroxylase 3	+	+	–
Transglutaminase II	+	+	–
α-SMA	–	–	–
***FDC markers***			
CD23	–	–	–
D2–40	–	–	–
Clusterin	–	–	–
CNA.42	–	–	–
NGFR, low affinity	–	–	–
CD21	–	–	–
CD35	–	–	–
***Histiocytic/dendritic cell markers***			
CD1a	–	–	–
CD11c	–	–	–
Langerin	–	–	–
DC-SIGN	–	–	–
DC-LAMP	–	–	–
CD83	–	–	–
DEC205	–	–	–
S-100	–	–	–
CD68	–	–	–
Fascin	+	+	–
Factor XIIIa	–	–	–
***Others***			
Melan A	–	–	–
HMB45	–	–	–
Desmin	–	–	–
Vimentin	+	+	–
CD34	–	–	–
CD117	–	–	–
ALK	–	–	–
EGFR	< 10%	20%	–
p53	95%	80%	–
Ki-67 (hot spot)	> 75%	15%	< 50%

– negative, ± partially positive, + diffusely positive, *PSA* Prostate-specific antigen, *NGFR* Nerve growth factor receptor, *FRC* Fibroblastic reticular cell, *FDC* Follicular dendritic cell, *EBER-1* (in situ hybridization) Epstein-Barr virus-encoded small RNA-1, *ALK* Anaplastic lymphoma kinase, *EGFR* Epidermal growth factor receptor

**Fig. 4:**
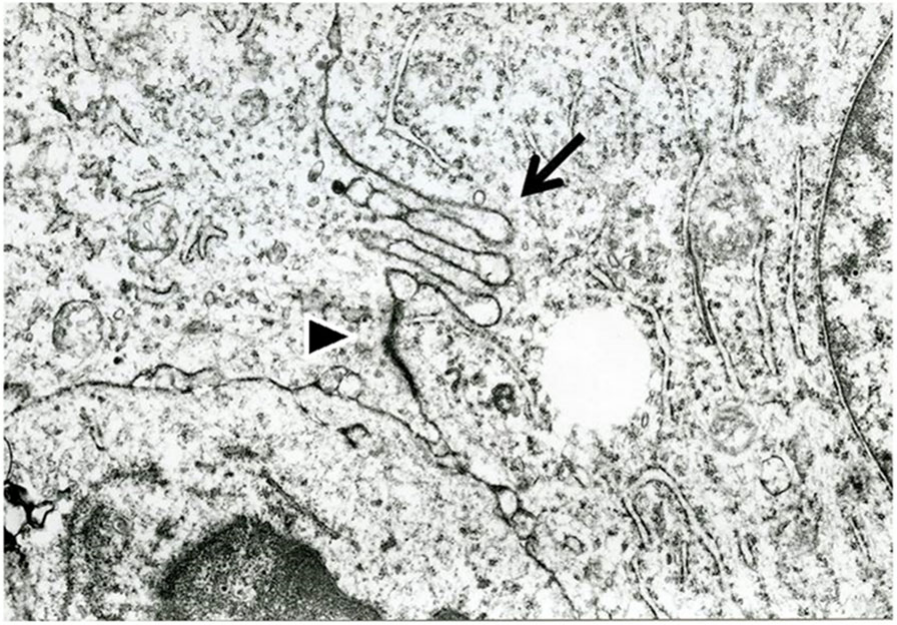
Electron microscopy analysis of primary cytokeratin-positive interstitial reticulum cell (CIRC) tumor cells. A few interdigitating cell processes were observed (arrow). Some tumor cells had desmosome-like junctions (arrowhead). Of note, the tumor cells had no endocrine granules. Original magnification, × 20,000

The ultrastructure of the tumor cells showed large cells with occasional indented nuclei with vesicular chromatin. The cytoplasm of the cells contained intermediate filaments. Some tumor cells exhibited dendritic interdigitating cytoplasmic processes and desmosome-like junctions (Fig. 4). The tumor cells, however, had no endocrine granules.

#### Autopsy findings

**Fig. 5:**
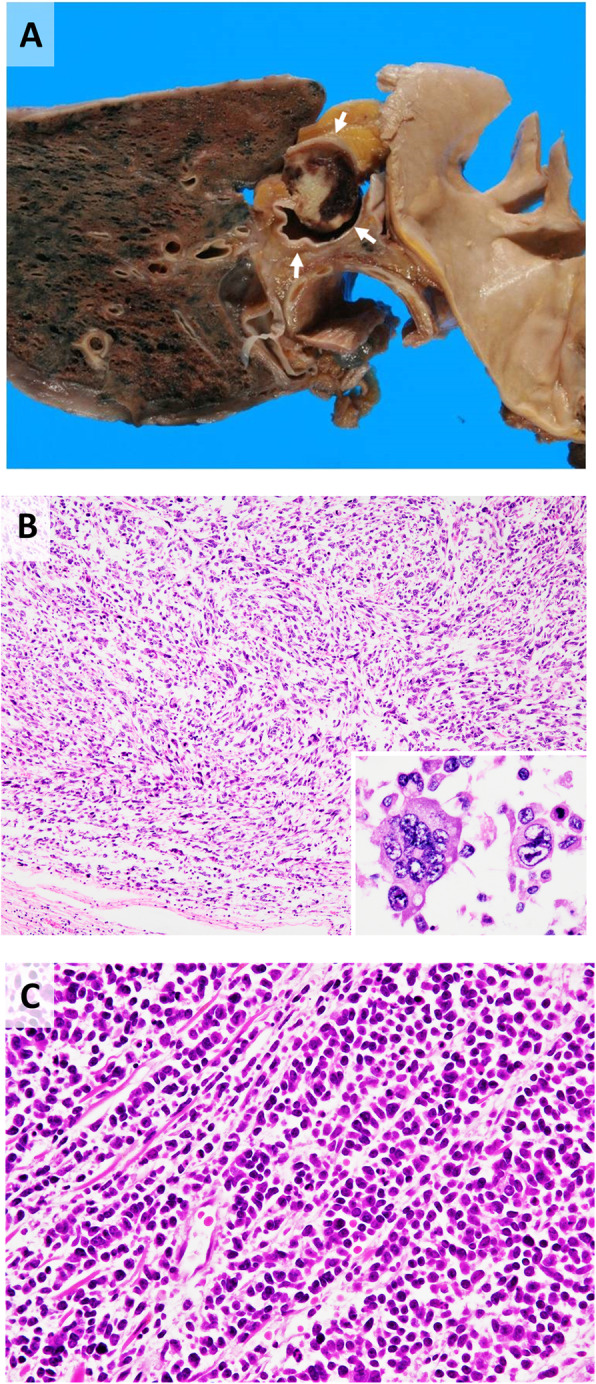
Relapsed cytokeratin-positive interstitial reticulum cell (CIRC) tumor and small cell carcinoma at autopsy. **a** Macroscopic findings of the CIRC tumor invading into the superior vena cava (arrow). **b** Microscopic findings of the CIRC tumor composed of spindle-shaped cells with a loose flow. **Inset**: high-magnification view of atypical multinucleated cells. **c** Small cell carcinoma showing typical histological findings on autopsy. Tumor cells had a high N/C ratio and formed focal molding structures. This finding was completely different from that of CIRC tumors

A relapsed CIRC tumor was located that extended from the apex of the right lung to the clavicle fossa. It had progressed to the superior vena cava (Fig. 5a) and was partially obstructing it. The patient presented with superior vena cava syndrome, which was the cause of his death. Histologically, changes in tumor cell morphology were observed, resulting in the formation of spindle cells that formed loose fascicles (Fig. 5b). The tumor cells showed extremely advanced pleomorphism in the relapsed tumor compared with that in the primary tumor, with numerous bizarre large and multinucleated cells and many typical and atypical mitoses (42/10 high-power fields). The atypical large cells showed greater increases in size and atypia in the relapsed tumor than in the primary tumor. The tumor cells were mixed with delicate collagen fibers. A massive area (approximately 80%) of necrosis was observed. The CIRC tumor had also metastasized to the right paratracheal lymph node.

The tumor cells of relapsed CIRC tumors were positive for CK8, CK18, CAM5.2, AE1/AE3, EMA, vimentin, fascin, l-caldesmon, prolyl 4-hydroxylase 1, lysyl hydroxylase 3, and transglutaminase II, indicating the presence of a CIRC tumor (Table 1, Fig. 3). Immunoreaction with cytokeratins showed dendritic staining patterns in tumor cells. In contrast to the primary lesion, the relapsed tumor was negative for CK7. The relapsed tumor cells were negative for NSE, chromogranin A, synaptophysin, CD56, Napsin A and ALK. They were also negative for prostate-specific antigen, NKX3.1 and P504S, thus ruling out metastatic adenocarcinoma of the prostate. EBER-1 ISH was negative. Twenty percent of the tumor cells showed weak expression of EGFR. Up to 80% of the tumor cells heterogeneously expressed the p53 protein. The Ki-67 LI was very low (15%).

A small cell carcinoma had formed in the hilar region of the right lung. Histologically, the tumor cells showed the typical finding, which is completely different from the morphology of CIRC tumors (Fig. 5c). Furthermore, immunohistochemical staining of TTF-1 and neuroendocrine cell markers (NSE, chromogranin A, synaptophysin, and CD56) were positive to varying degrees (Fig. 3). Immunohistochemical staining was negative for FRC markers, which are useful for diagnosing CIRC tumors (Fig. 3). Staining of Napsin A, ALK, and EGFR were negative. The tumor metastasized to the liver, left paratracheal lymph node, and tracheal bifurcation lymph node. The small cell carcinoma and the CIRC tumor were located somewhat close to each other in the hilar region, but there was no clear continuity between them.

In addition, a small adenocarcinoma was found in the left lobe of the prostate. The adenocarcinoma corresponded to a Gleason pattern of 3 + 4, and it had remained within the prostate. There was no lymphatic or vascular invasion. The tumor cells were diffuse and strongly positive for prostate-specific antigen, and the focal cells were weakly positive for NKX3.1.

## Discussion and conclusion

The first report of CIRCs in normal lymph nodes was published in 1987 by Franke and Moll [15]. CIRCs are a subset of FRCs that express vimentin as well as CK8 and CK18 [15–17]. Alpha-SMA is expressed in 20–60% of CIRCs, whereas desmin is expressed in 1–10% of CIRCs [8, 16]. CIRCs have elongated cell processes and are found in the extrafollicular compartment of the outer and inner cortex along blood vessels. They have also been observed in the spleen and tonsils. Electron microscopy has confirmed the presence of abundant intermediate filaments and tonofilament-like tight bundles in CIRCs. Since Gould et al. first reported on CIRC-derived tumors in 1990, to the best of our knowledge, only seven relevant articles have been published in English [8–14]. A total of 12 cases have been diagnosed as CIRC tumors, including our case. Although one case was reported to involve an FRC tumor, the tumor recurred and morphed into a CIRC tumor [11]. Table 2 summarizes the clinical data of published CIRC cases. The age of the patients in these reports ranged from 21 to 75 years, and the subjects were predominantly male. Half of the cases involved tumors that were localized in the hilum and mediastinal lymph node. Tumors ranged in size from 0.9 to 9 cm. The maximum size was observed for a relapsed tumor. The histopathological findings were variable, but on the basis of morphological features, the published cases were divided into three types. The initial type mainly involved spindle cells that formed fascicles, and the storiform pattern often predominated. As a result, the tumor in this type gave the impression of a noncohesive and reticular lesion. The second type mainly involved nests of round or oval cells, and a vaguely nodular pattern predominated. The tumor may also have had a cohesive and epithelioid-like appearance. The third type involved the admixture of the two aforementioned patterns; therefore, the proliferative pattern was mixed and variable. All three types had the common feature of involving tumor cells that were mixed with numerous lymphocytes, granulocytes, and plasma cells. Occasionally, multinucleated tumor cells and large or giant tumor cells were admixed. According to previously reported cases, the spindle cell type is less frequently observed than the other two types. In the present case report, the primary tumor showed a round or oval cell (epithelioid cell) type, but the recurrent tumor morphed into the spindle cell type, indicating the transformability of neoplastic cells.

**Table 2:** Clinical data of published CIRC tumors

Case No.	Diagnosis	Complication (malignancy)	Age	Sex	Nodal / Extranodal	Region	Size	Treatment	Outcome	References
1	CIRC tumor		57	M	Nodal	Left hilum	NA	A left thoracotomy Radiotherapy; 50 Gy	Alive and well, 9 years	Eur J Cancer 1990; 26: 1121–6
2	CIRC tumor		44	M	Nodal	Left hilum	4 cm	A left thoracotomy	Alive and well, over 3 years	
3	CIRC tumor		61	M	Nodal	Right hilum	NA	A right thoracotomy Radiotherapy; 50 Gy	Alive and well, 13 months	
4	CIRC tumor		21	F	Nodal	Mediastinal (superior)	2.5 cm	CHOP chemotherapy Radiotherapy; 21 Gy VP-16 and CBDCA chemotherapy	Died of disease, 9 months	Am J Surg Pathol 2000; 24: 107–16
5	CIRC tumor		73	F	Extranodal	Right proximal forearm	NA	Surgery (excision)	NA	
6	CIRC tumor		62	M	Nodal	Mediastinal (posterior)	7.4 cm	Radiotherapy; 65 Gy	NA	
7	CIRC tumor		66	M	Nodal	Submandibular	1.5 cm	Surgery (excision) Radiotherapy	Alive and well, 12 months	Histopathology 2003; 43: 491–4
8	FRC tumor (primary)		70	F	Nodal	Right submandibular	3.0 cm	Surgery	Died of disease, 10 months	Histopathology 2003; 43: 583–91
	CIRC tumor (relapse)				Nodal	Local relapse	9.0 cm	VACOP B chemotherapy Two cycles of CNOP chemotherapy		
9	CIRC tumor	Rectal adenocarcinoma	62	M	Extranodal	Spleen	0.9 cm	Surgery (low anterior resection, splenectomy)	Alive and well, 2 years	Int J Surg Pathol 2014; 22: 447–50
10	CIRC tumor with FDC features		54	F	Nodal	Right axillary	NA	Surgery (excision)	Alive and well, 2.5 years	Am J Surg Pathol 2015; 39: 573–80
11	CIRC tumor	Uterine endometrioid carcinoma	67	F	Nodal	Pelvic	3.5 cm	Surgery (hysterectomy, bilateral adnexectomy, pelvic lymphadenectomy)	Alive and well, 9 months	Hum Pathol 2016; 49: 15–21
12	Current case (primary)		75	M	Nodal	Mediastinal (superior)	2.7 cm	Surgery (excision) CBDCA and DOC chemotherapy	Died of disease, 29 months	
	Current case (relapse)	Small cell lung carcinoma Prostatic latent carcinoma			Extranodal	Right lung apex to the clavicle fossa	1.5 cm	CDDP and VP-16 chemotherapy Three cycles of NGT chemotherapy Radiotherapy; 30 Gy		

*CIRC* Cytokeratin-positive interstitial reticulum cell, *FRC* Fibroblastic reticular cell, *FDC* Follicular dendritic cell, *CHOP* Cyclophosphamide, doxorubicin, vincristine, and prednisone, *VP*-*16* Etoposide, *CBDCA* Carboplatin, *VACOP-B* Etoposide, doxorubicin, cyclophosphamide, vincristine, prednisone, and bleomycin, *CNOP* Cyclophosphamide, mytoxantrone, vincristin, and prednisone, *DOC* Docetaxel, *CDDP* Cisplatin, *NGT* Nogitecan, *NA* Not available

The immunohistochemical profile of the 12 CIRC tumor cases is summarized in Table 3. All CIRC tumor cases were positive for epithelial markers, such as CAM5.2 (CK8), CK18, AE1/AE3 and EMA, and vimentin. The expression of desmin and α-SMA was variable. Two cases showed aberrant expression of S-100 protein and CD68 [10, 13, 14]. As a rule, staining of FDC markers, including CD21 and CD35, were negative. Only one case, which was reported as involving a CIRC tumor with features of FDCs, showed weak CD21 positivity [13]. In the present case, tumor cells coexpressed cytokeratins and vimentin, and they were positive for some FRC markers, including prolyl 4-hydroxylase 1, lysyl hydroxylase 3 and transglutaminase II [18, 19]. Chemotherapy and autopsy-related artifacts could be responsible for the differences in the morphologies of the tumors. In fact, there was a decrease in the Ki-67 LI of the relapsed tumor compared with that of the primary tumor. This suggested that the chemotherapy effect and autopsy-related artifacts occurred, because the autopsy sample showed 80% necrosis. Electron microscopy analysis was performed in nine cases, including our case, and in all cases, the tumors demonstrated some epithelial cell features, such as tonofilaments, desmosomes, and desmosome-like junctions. Tonofilaments are a feature of normal CIRCs. In fact, Gould et al. suggested that CIRCs show increased epithelial differentiation, including the formation of desmosomes, as they become neoplastic [8].

**Table 3:** Immunohistochemical results of published CIRC tumors

Author(s)	Primary/Relapse	CAM5.2 (CK8)	CK18	AE1/AE3	EMA	Vimentin	Desmin	α-SMA	S-100	CD21	CD68	p53
Gould et al. [8]		+	+	+	+	+	–	+	–	ND	ND	ND
Gould et al. [8]		+	+	+	+	+	+	+	–	ND	ND	ND
Gould et al. [8]		+	+	+	–	+	+	+	–	ND	ND	ND
Chan et al. [9]		+	+	+	+/−	+	–	–	–	–	–	ND
Chan et al. [9]		+	+	–	–	+	+	+	–	–	–	ND
Chan et al. [9]		+	+	+	+/−	+	–	–	–	–	–	ND
Schuerfeld et al. [10]		+	+	+	–	+	+	ND	+	–	+	ND
Lucioni et al. [11]	*primary	–	ND	–	–	+	–	+	–	–	+	20%
	relapse	+	ND	+/−	–	+	–	–	–	–	–	> 60%
Karim et al. [12]		ND	ND	+	ND	ND	+	+	–	–	ND	ND
Goto et al. [13]		+	ND	+	+/−	ND	–	–	+/−	+/−	–	ND
Bösmüller et al. [14]		+	ND	+	–	+	–	–	–	–	+	+/−
Current case	primary	+	+	+	+	+	–	–	–	–	–	95%
	relapse	+	+	+	+	+	–	–	–	–	–	80%

- negative, +/− often/partially positive, + positive, * primary tumor was diagnosed as fibroblastic reticulum cell tumor; *ND* Not done

CIRC tumors exhibit a wide spectrum of clinical behavior, which can result in outcomes that range from good to poor. Currently, there is no fixed treatment method for CIRC tumors. Surgical resection, chemotherapy, and radiotherapy are conducted, sometimes in combination. Including the present patient, three patients have died of the disease. The survival period ranged from 9 to 29 months after the initial diagnosis (Table 2).

The differential diagnoses, including FDC sarcoma, IDC sarcoma, and metastatic carcinoma, are important. Binucleated or multinucleated tumor cells are often seen in FDC sarcoma that are similar to those in CIRC tumors. The distribution pattern of tumor cells in lymph nodes and the absence of collagen fibers mixed with tumor cells in FDC sarcoma are findings that are different from those in CIRC tumors. Similar to CIRC tumors, IDC sarcoma tumor cells present in a paracortical distribution with residual follicles. Unlike CIRC tumors, IDC sarcoma does not present with interspersed delicate collagen fibers intermingled with tumor cells. Usually, in metastatic carcinoma, there is no diffuse spreading of small lymphocytes, plasma cells or eosinophils throughout the tumor, and the cell border is clear. Metastatic carcinoma presents in the lymph node without the preservation of follicles. The nuclei of the CIRC tumor are more highly vesicular and show more finely dispersed chromatin and a thinner nuclear membrane than metastatic carcinoma cells. However, the histological appearance of CIRC tumors is sometimes indistinguishable from that of FDC sarcoma, IDC sarcoma, and metastatic carcinoma. Therefore, immunophenotyping of markers, such as CD21 and S-100 protein, is necessary for precise diagnosis. In the case of metastatic carcinoma, it is essential to investigate primary lesions. In our case, findings similar to those of Hodgkin lymphoma were found in the primary tumor, and differentiation was required. Compared with that in Hodgkin lymphoma, in CIRC tumors, the number of large tumor cells is much higher than that of lymphocytes. The tumor cells tend to proliferate, which preserved the lymphoid follicle, and this was not observed in Hodgkin lymphoma. Ultimately, the CIRC tumor was distinguished from Hodgkin lymphoma based on the immunostaining results. In contrast, careful examination was required because the patient suffered from small cell lung carcinoma after the onset of the CIRC tumor. Hematoxylin-eosin staining showed that the cell morphology of the CIRC tumor and small cell carcinoma were clearly different. However, evolution of small cell carcinoma towards sarcomatoid tumors following therapy must be distinguished from recurrent CIRC tumors with caution. Because CIRC tumors are similar to small cell lung carcinomas and express epithelial markers such as cytokeratin, more detailed examination using immunohistochemical and ultrastructural analysis is essential for differential diagnosis. Immunohistochemically, CIRC tumor cells showed positivity for FRC markers and negativity for neuroendocrine markers, whereas small cell lung carcinoma shows the opposite staining pattern. Ultrastructurally, CIRC tumor cells had no endocrine granules, and dendritic interdigitating cytoplasmic processes were rarely observed.

The pathogenesis of CIRC tumors is unknown; however, CIRCs grow from various reactive and inflammatory neoplastic lymphadenopathies and show an atypical appearance in human immunodeficiency virus-associated lymphadenopathy [20]. Little data are available on the expression of p53 in dendritic cell tumors [21–23]. Chan et al. suggested a role for p53 in the malignant transformation of dendritic cells in Castleman disease associated with FDC sarcoma. Although the relationship between CIRC tumors and p53 expression is unclear, Lucioni et al. reported increased p53 expression in relapsed tumors [11].

In conclusion, we first demonstrated here the transformation of CIRC tumor morphology from an epithelioid cell type to a spindle cell type and compared the primary lymph node lesion and the recurrent lesion on autopsy.

## Supplementary information

**Additional file 1: Fig. A.** Histology of prostatic adenocarcinoma at autopsy. The tumor cells formed irregular small glands with small nests. The tumor margin is indicated by *arrows*. **Fig. B.** Immunohistochemical staining of small cell lung carcinoma using the p53 antibody. The tumor cells showed no positive staining in the nuclei. **Fig. C.** Immunohistochemical staining of colon carcinoma using the p53 antibody as a control. In contrast to small cell carcinoma, colon carcinoma showed positive staining in the nuclei.

## Data Availability

The data and materials are available upon request from the corresponding author.

## Abbreviations

FDC: Follicular dendritic cell
IDC: Interdigitating dendritic cell
FRC: Fibroblastic reticular cell
CIRC: Cytokeratin-positive interstitial reticulum cell
CK: Cytokeratin
EMA: Epithelial membrane antigen
α-SMA: Alpha smooth muscle actin
EBV: Epstein-Barr virus
LI: Labeling index
