# Supplementary figures and images for: Cytokeratin-positive interstitial reticulum cell (CIRC) tumor in the lymph node: a case report of the transformation from the epithelioid cell type to the spindle cell type
Source: Diagn Pathol. 2020 Sep 26;15:121. doi: 10.1186/s13000-020-01032-9 (PMC7519525; doi:10.1186/s13000-020-01032-9)

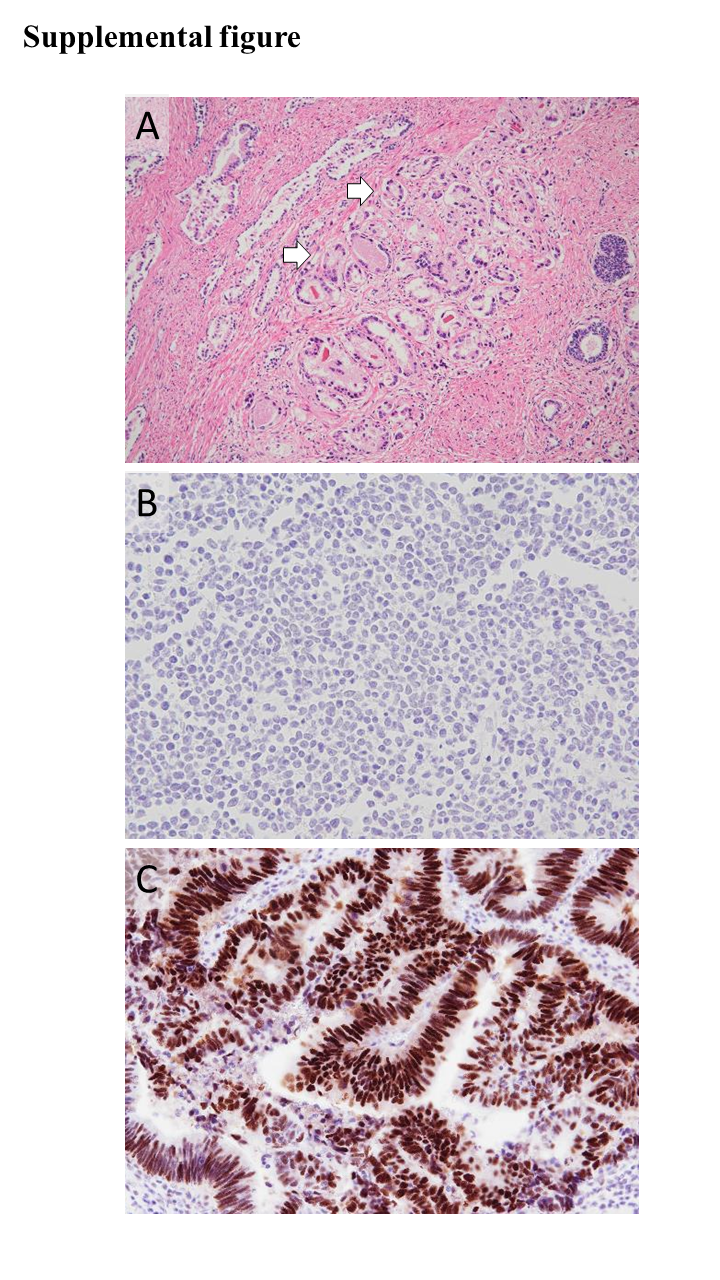

Supplement: Supplementary file 1 — Additional file 1: Fig. A. Histology of prostatic adenocarcinoma at autopsy. The tumor cells formed irregular small glands with small nests. The tumor margin is indicated by arrows. Fig. B. Immunohistochemical staining of small cell lung carcinoma using the p53 antibody. The tumor cells showed no positive staining in the nuclei. Fig. C. Immunohistochemical staining of colon carcinoma using the p53 antibody as a control. In contrast to small cell carcinoma, colon carcinoma showed positive staining in the nuclei. [file 13000_2020_1032_MOESM1_ESM.tif]
